# Should I stay or should I go? The emergence of partitioned land use among human foragers

**DOI:** 10.1371/journal.pone.0218440

**Published:** 2019-07-11

**Authors:** Jacob Freeman, John M. Anderies, Raymond P. Mauldin, Robert J. Hard

**Affiliations:** 1 Anthropology Program and Ecology Center, Utah State University, Logan, UT, United States of America; 2 School of Sustainability, Arizona State University, Tempe, AZ, United States of America; 3 Center for Archaeological Research, University of Texas at San Antonio, San Antonio, TX, United States of America; 4 Department of Anthropology, University of Texas at San Antonio, San Antonio, TX, United States of America; Max Planck Institute for the Science of Human History, GERMANY

## Abstract

Taking inspiration from the archaeology of the Texas Coastal Plain (TCP), we develop an ecological theory of population distribution among mobile hunter-gatherers. This theory proposes that, due to the heterogeneity of resources in space and time, foragers create networks of habitats that they access through residential cycling and shared knowledge. The degree of cycling that individuals exhibit in creating networks of habitats, encoded through social relationships, depends on the relative scarcity of resources and fluctuations in those resources. Using a dynamic model of hunter-gatherer population distribution, we illustrate that increases in population density, coupled with shocks to a biophysical or social system, creates a selective environment that favors habitat partitioning and investments in social mechanisms that control the residential cycling of foragers on a landscape. Our work adds a layer of realism to Ideal Distribution Models by adding a time allocation decision process in a variable environment and illustrates a general variance reduction, safe-operating space tradeoff among mobile human foragers that drives social change.

## Introduction

The Texas Coastal Plain (TCP) contains some of the oldest and longest used hunter-gatherer mortuary sites in the Americas. Hunter-gatherer mortuary locations, rates of burial and rates of grave good deposition on the TCP all provide deep-time records with enormous potential to investigate how foraging populations distribute on a landscape [1–9]. Researchers on the Texas Coastal Plain have long argued that the development of a hunter-gatherer mortuary complex between 7000 and 750 cal BP reflects specialization in the use of particular resource zones–habitat partitioning–and the development of territorial ownership–making entrance into a habitat more costly for some individuals [1–9]. By 7,000 cal BP, TCP foragers established small cemeteries centered on the use of resources from the Gulf of Mexico and inland along freshwater streams and uplands [1, 2]. Around 3,000 cal BP, cemeteries expanded quickly, peaking in the number of locations and the number of interred individuals between 1,000 and 750 cal BP (page 137 in [1]) (see also [3–5, 7–9]). Importantly, during this time-period, human bone isotope evidence indicates a restricted use of resources within well delineated habitat types (e.g., riverine savanna vs. coastal estuary) [2, 10]. Why? What mechanisms led foragers to partition their use of space on the prehistoric TCP, and how might the process of partitioning relate to the adoption of territorial ownership?

In this paper, we build and analyze a spatially explicit model of coupled space use and resource dynamics to investigate this question. The model allows us to isolate the social-ecological conditions that may cause a population of foragers to partition in space and time, focusing on a narrower range of habitats. The model describes the distribution of foragers as individuals respond to seasonal changes in the availability of resources, as well as longer-term changes in climate and population. The model that we build is more complex than typical foraging models used by anthropologists (e.g., [11–13] because the model describes important feedback relationships between resources, individual foraging and land use; however, the model is also less complex than agent based models that describe more details of particular systems (e.g., [14–16]). The model makes operational a general dynamic theory of hunter-gatherer population distributions in space and illustrates a variance reduction, safe-operating space tradeoff. A variance reduction, safe-operating space tradeoff occurs when individual decisions to maximize the consistency of the supply of food in the short-run creates a social-ecological system vulnerable to disruption in the longer-run. Such trade-offs, we propose, provide one mechanism that causes hunter-gatherers to partition in space and adopt more labor intensive institutions and technologies.

### A spatially explicit population-resource model

**Fig 1:**
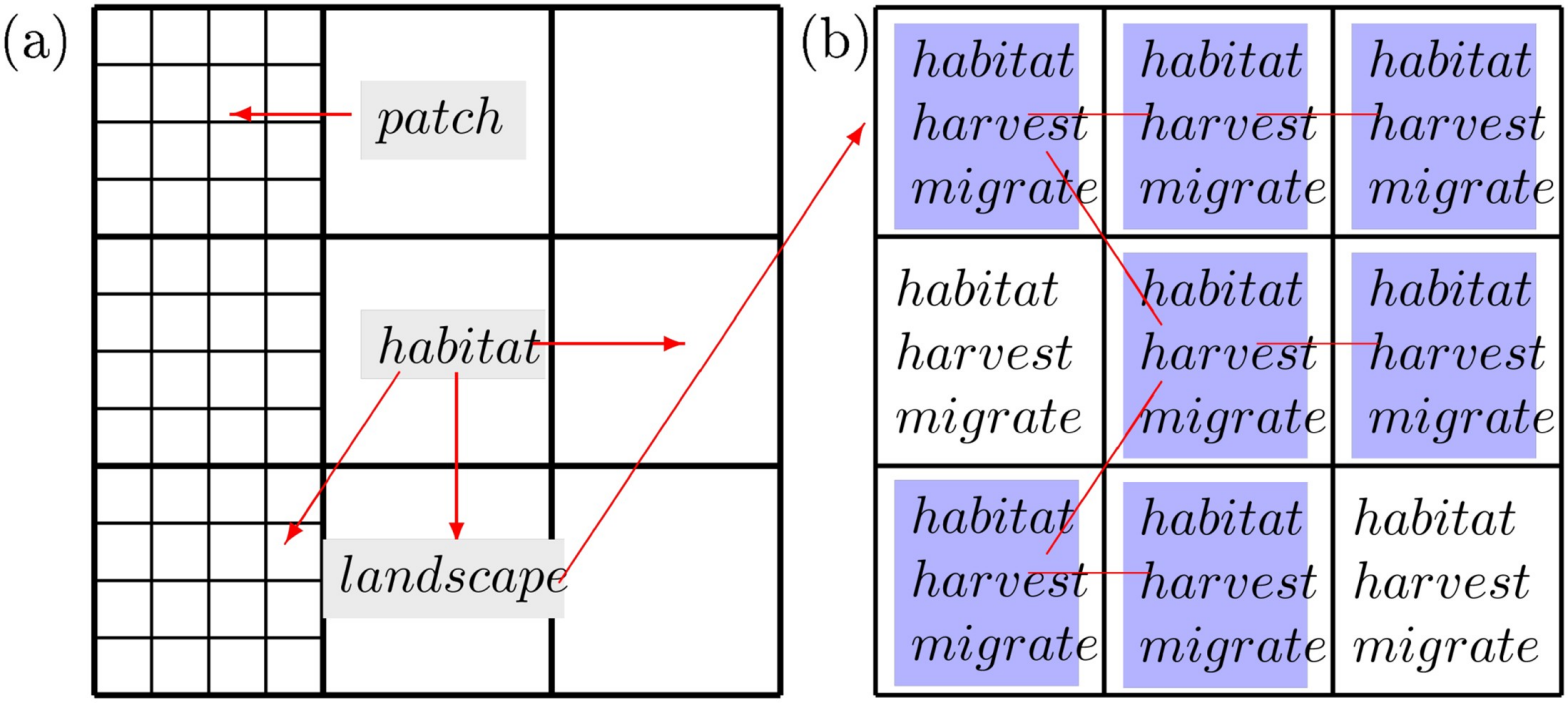
Conceptual landscapes. (a) Hierarchy of foraging decisions in space [20]. The far left column represents patch choices. Patch choices occur daily or weekly as foragers radiate out from base camps to access the smallest patch blocks. Habitat choices occur monthly-to-annually as foragers choose between the moderate sized blocks, and, at the landscape scale, foragers make decisions about their home-ranges a few times over decades by choosing between blocks of landscape (e.g., pages 380–383 in [21]). (b) Habitats that foragers may use. Foragers must choose where to reside (habitat), how much time to spend in a given habitat (harvest) and which set of habitats to use (migrate). These decisions interact with each other by affecting resources and the decisions of other foragers. The set of blue habitats comprise a forager’s “realized territory.” We simplify our model by studying a two habitat landscape.

Fig 1 illustrates the conceptual foundation of our investigation. Fig 1a represents a decision hierarchy that human foragers face in their use of space, and Fig 1b illustrates the scale of the hierarchy relevant to the emergence of habitat partitioning and territorial ownership. In general, at the habitat scale, some set of potential habitats exists on a landscape in which foragers may reside. Each forager must decide how many of that potential set of habitats to use, how much time to spend in any given habitat exploiting its patches and which habitats to move between. Our basic idea is that partitioning and ownership interrelate due to the feedback between these decision processes in a variable environment. To study the feedback between these decision processes, we construct a dynamic ecological model called the spatial foraging effort model (or the SPDm for short). The SPDm is a two habitat model. This allows us to hold the question of which habitats to move between constant and focus on the feedback between the number of potential habitats (one vs. two), and how much time to spend in a given habitat exploiting its resource patches. The logic of the SPDm extends two classic models form Foraging Theory, Ideal Distribution Models (IDMs) [17] and the Marginal Value Theorem (MVT) [18, 19].

First, to understand differences in the population distribution of foragers, IDMs link individual decisions about where to locate in space with a populations’ distribution on a landscape [17]. Two common applications of IDMs to human populations include the Ideal Free and Ideal Despotic Distributions (e.g., [12, 22–24]). Both models assume that individuals locate in the habitat where they maximize their fitness, at any given moment in time, relative to other habitats. The main difference between the two models is that the Ideal Free Distribution assumes that foragers have complete freedom to move between habitats, while the Ideal Despotic Distribution assumes territorial ownership. Territorial ownership refers to rules and norms that restrict entry into a habitat, making entrance more costly due to the existence of social norms or attacks [25]. In the Ideal Despotic Distribution, ownership crowds individuals out of higher ranked habitats, pushing population densities up in lower ranked habitats above what one would expect from the Ideal Free Distribution [17]. While comparing the Ideal Free and Despotic models help us understand how territorial ownership might change the distribution of population density on a landscape, the models do not help us understand why partitioning and territorial ownership would emerge in the first place.

Specifically, IDMs have a single long-run average solution (equilibrium). The simplicity of a single long-run average solution gives such models a high degree of generality, but also glosses over an important process. If there are identifiable cultural groups on a landscape, then IDMs actually allow them to distribute in at least two indistinguishable ways: (1) Foragers may cycle and completely mix in space and time, or (2) foragers may partition in space so that the average density over the whole space and over time is an IDM. For example, the application of the Ideal Free Distribution to explain the distribution of foragers in modern fisheries illustrates that stable and equal return rates in alternative fishing habitats can either emerge from multiple boats (individuals) cycling through habitats (mixing in space and time), or the same boats constantly fishing the same habitat (partitioning in space) [26]. In principle, the same observation applies to the Ideal Despotic Distribution. Individuals who enforce ownership might partition, or they might cycle between habitats, for instance, following seasonal changes in resources, simply crowding-out less territorial individuals in each habitat that they visit. This would resemble what Mancur Olsen famously called roving bandits [27].

A classic ethnographic example of high rates of forager cycling comes from the Kalahari, where groups of families associate with resources around particular water holes, but the composition of groups within a particular territory (n!ore) fluxes as individuals and nuclear families move in and out [28, 29]. Often this flux occurs through reciprocal visiting. “Visitors join residents in the exploitation of resources, and the days take is unobtrusively distributed within the camp at the days end…No matter where they are from, as long as people are living together in a single camp the n!ore’s food is theirs to share” (page 333 in [30]). Conversely, among the Modoc of modern day California, territorial “boundaries were precisely defined and understood by the Modoc and transgression meant war” (page 201 in [31]). Families were self-sufficient and tied to particular tracks of territory, with only the sick and elderly experiencing the freedom to move across boundaries with the ease of Kalahari foragers. Either pattern, rapid cycling (!Kung) or slow cycling (Modoc), could lead to an ideal distribution in which the mean fitness (or a proxy for fitness like return rate) of individuals is equal, in the long-run, among alternative habitats.

In the end, IDMs merely describe how the density of mobile resource users partitions according to immobile resource densities. IDMs do not tell us which forager is where, because such models do not label the foragers. Thus, IDMs cannot resolve cycling (rapid vs. slow). Put in terms of ideal distributions, why might the form that ideal distributions take, either rapid forager cycling or habitat partitioning (slow cycling), change over time? To answer this question, we must understand the interrelationship between population distributions in aggregate, and habitat choices at the level of individual agents.

Second, the Marginal Value Theorem (MVT) [18, 19] describes habitat (or patch) choices. The MVT illustrates that a solitary forager should stop harvesting resources within a habitat when the net rate of food intake within the habitat drops to the average for the landscape overall (in our model, a landscape of two habitats). Importantly, this insight depends on the assumption that a forager does not revisit a habitat, and that the harvest behaviors of other foragers do not affect the availability of resources (pages 251-252 in [19]). However, when foragers revisit habitats and their behaviors interact through depletion, information on the state of resources among alternative habitats becomes critical for making land use decisions [19, 32]. Furthermore, foragers may experience non-linear, threshold changes in the quality of particular habitats [32–36].

For example, any given habitat on a landscape may be modeled as a forager-resource system that settles into regimes that approximate stable equilibria. In such a model, Freeman and Anderies illustrate how either increasing population density or decreasing the growth rate of resources within a single habitat makes that modeled forager-resource system vulnerable to a flip from a productive equillibrium into a poverty trap equilibrium [32]. This is important. On a landscape with habitats that vary in productivity due to migrating foragers, the emergence of habitats that are either potentially productive or a poverty trap for individual foragers can have a feedback on the decision making of individuals in ways that the MVT does not investigate. Freeman and Anderies argue that when it is necessary and difficult to compute which habitats might flip into a poverty trap, this should provide a proximate incentive to adopt more exclusive ownership rules [25, 32].

In short, as in the MVT, the SPDm models foragers who adjust their time within a habitat based on expected average returns from all habitats on a landscape. In addition, the SPDm describes a more realistic situation than the MVT: Foragers constantly face a tension between the benefits of moving to a new habitat (a more consistent intake of resources and information on that habitat) and the costs of allowing other foragers to enter a ceded habitat (depletion of the resources and loss of information). The form that ideal distributions of foragers takes (rapid cycling vs. partitioning), we suspect, follows from this tension inherent in movement to create larger vs. smaller realized territories.

### Model structure and assumptions

**Fig 2:**
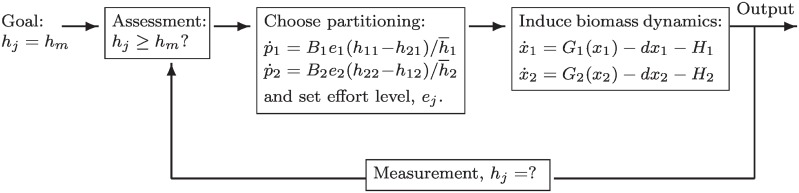
Basic block, feedback diagram of the SPDm. Equations defined in the Model and methods section. The diagram reads as follows: The goal is set outside the loop, and enters from the left. The feedback loop iterates as individuals assess whether the goal is met. Foragers adjust their effort and spatial partitioning, which affects biophysical dynamics and produces an output (harvest). Foragers then measure (do I feel hungry) and compare the harvest with their goal in the next time period.

We provide an intuitive description of the model’s assumptions here, and we present the formal equations in the Model and methods section. Fig 2 summaries the SPDm. In this model, a forager has the goal of obtaining a harvest of energy (*h*_*j*_) equal to a baseline energy target (*h*_*m*_) necessary for reproducing social relationships, maintaining biological function and reproducing biologically. In order to achieve this goal, a forager chooses how much time to spend harvesting resources from either habitat one or habitat two, which are both affected by harvest pressure and an external driver of productivity (e.g., rainfall). The harvest of resources from habitats one and two, in turn, affects how much time foragers spend in a given habitat (i.e., whether a forager cycles between the two habitats). Choices about harvest and whether to cycle between habitats results in an output of resources, which foragers then assess against their baseline target and, again, make choices about harvesting and partitioning. This system captures three fundamental processes.

**Fig 3:**
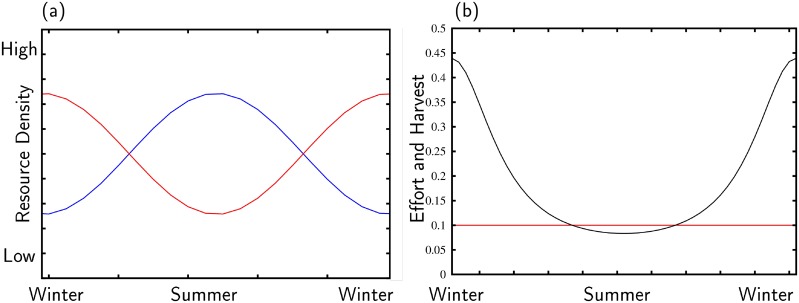
. Graph (a): Resource dynamics. Red curve (*x*_1_) illustrates resource variation of the TCP coast and the blue curve (*x*_2_) resource variation inland in riverine areas of the TCP. Graph (b): Harvest dynamics of a partitioned forager in the riverine area of the TCP (*x*_2_): Harvest (red curve) and foraging effort (black curve).

First, there are two habitats in which resources grow (*G*(*x*_*i*_)) and deplete due to decay ($d_{x_{i}}$) and harvest pressure (*H*_*i*_). Importantly, the resources in each habitat vary periodically due to external drivers (seasonality). Here we assume that variation in the productivity of the habitats remains out of phase. Resources in one habitat peak during the winter (e.g., the Texas Coast) and resources in the other habitat peak during the summer (e.g., inland gallery forests on the Texas Coastal Plain). Fig 3a illustrates this dynamic. Note that the peaks of the red and blue curves are offset. The assumption that resources vary 180 degrees out of phase fits the resource structure of the Texas Coastal Plain. Please note that qualitatively our results hold for a system in which resources are 90 degrees out of phase as well.

Second, two representative agents occupy the habitats. One agent represents the mean forager in group one, and the second agent represents group two. Group one has an association with habitat one and group two with habitat two. These associations follow the convention that mobile foragers form home-ranges. However, the association is fluid and does not represent a cost for other foragers who may move into the habitat (e.g., [30, 37]), which contrasts with such costs generated by territoriality [38].

Each forager makes two types of decisions: How much time to spend harvesting within a given habitat and whether to use multiple habitats (one or two?). The decision about how much to harvest now in habitat *i* is defined by a satisfying process. Foragers simply ask: Have I met *h*_*m*_? If the answer is yes, foragers use their excess time to bond socially, rest, etc. If the answer is no, foragers spend their time harvesting resources (see Eqs 3–7, Model and methods section). Fig 3b illustrates how this dynamic works, in particular, when forcing a forager to remain in one habitat. The black curve represents time spent harvesting resources and the red curve harvest (*h*_*j*_) in habitat two (*x*_2_, the blue curve on Fig 3a). Note that as productivity increases (the blue curve on Fig 3a rises), work effort declines (the black curve on Fig 3b). The red curve in Fig 3b remains constant because, in this case, the partitioned forager maintains a constant level of harvest by adjusting her level of foraging effort up and down with the productivity of the habitat.

Finally, the benefits of residential cycling between habitats include the acquisition of information and a potentially more consistent supply of resources [39–42]; however, this comes with the opportunity cost of leaving known resources behind. We capture this tension between known and unknown resources by the equations that govern the degree of partitioning or how rapidly foragers mix in space and time (Eqs 8–10, Model and methods section). A forager changes the proportion of her time budget in a given habitat by taking into consideration her current proportion of time in a given habitat and the normalized difference in harvest per unit effort between the two possible habitats. This process captures the following heuristic: ‘Does changing my proportion of time in a habitat affect my harvest?’ If yes, then a forager is more willing to change the proportion of time they spend in a given habitat. Willingness to change, however, also depends on how well a forager knows the other habitat. If a forager spends 90% of her time in habitat one, then she does not know much about habitat two. This uncertainty reduces the willingness to change the proportion of time in habitat one. When the proportion of time in habitat one is 50%, foragers know both habitats well, and are more willing to change their location. In short, information about alternative habitats and the difference in harvest per unit effort between alternative habitats interact. When foragers cycle between habitats and completely mix in space, information remains abundant and the opportunity costs of leaving known resources low. Thus differences in harvest per unit effort more strongly influence decisions.

In sum, we have a two habitat system described by four ordinary differential equations. In this system, the change in biomass, ${\dot{x}}_{i}$, effort devoted to foraging for resources, *e*_*j*_ and the change in the proportion of time spent in a given habitat ${\dot{p}}_{j}$ co-evolve. These equations capture the interplay between uncertainty in the productivity of resources, decision making about how much time to spend collecting resources and how many habitats to use. The distribution of foragers in space emerges from the interplay of these processes.

## Model results

Here, we summarize our key finding and, in the following subsections, illustrate the dynamics of the model that buttress each result. Our analysis illustrates trade-offs and the existence of multiple regimes of land use. We find that rapid forager cycling is a highly effective regime of land use—raises carrying capacity—but also sets a system up for collapse into partitioned groups that potentially live in a “Malthusian Purgatory.” Specifically,

1. All else equal, above a critical threshold that sets the severity of seasonal changes in resources, rapid forager cycling has a higher maximum population density than habitat partitioning. Below this threshold, habitat partitioning results in a higher maximum population density.
2. Increases in the maximum population density at which foragers maintain a consistent level of calorie intake comes with tradeoffs. For example, in aseasonal environments, partitioning increases the maximum population density at which foragers can maintain a consistent intake of calories. However, partitioning in space requires giving-up information about opportunities to find other fitness enhancing resources and, importantly, makes foragers more vulnerable to lower frequency (decade-to-century scale) perturbations. The implication: Longer-term variation in resources favors cycling between habitats.
3. Forager-resource systems experience a general variance reduction, safe-operating space tradeoff. This means that forager cycling increases the carrying capacity of the environment, reducing variation in the intake of calories for individuals. However, as forager populations approach the carrying capacity of a given social-technological set of strategies, the ability of the ecosystem and social system to withstand perturbations declines. As a consequence, foragers become vulnerable to cascades of resource failure as they cycle between habitats. This dynamic should favor, in some environments, the adoption of territoriality, and partitioning should emerge from territoriality.

### Results 1 and 2: The advantages of forager cycling in variable environments

**Fig 4:**
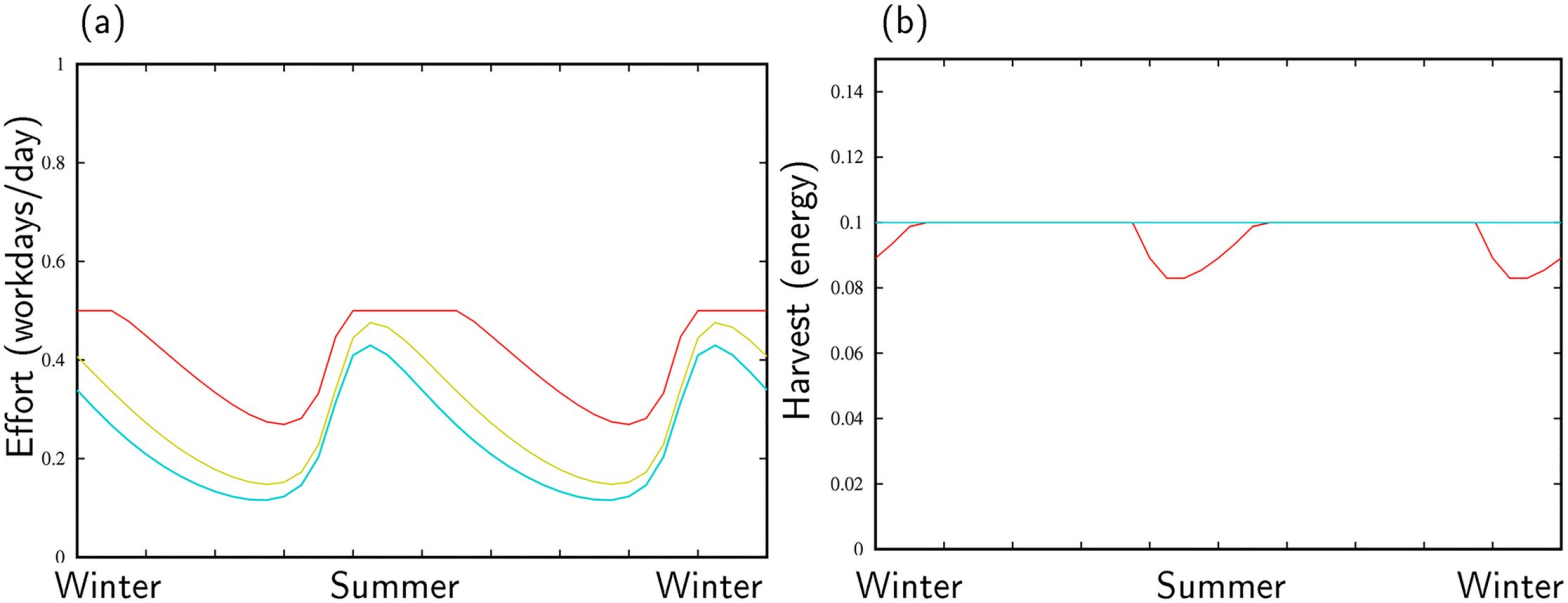
Foraging effort (a) and harvest (b) over a two-year period in habitat one for increasing population levels when foragers partition. Blue line–*N*_*i*_ for all i = 1; yellow-green line–*N*_*i*_ for all i = 2; Red line–*N*_*i*_ for all i = 3.5. The dynamics in habitat 2 are identical. Note that the resource growth rate, *r*, and the minimum harvest level *h*_*m*_, simply set the units for (or scale the) sustainable population size. Our choice to set *h*_*m*_ = 0.1, sets the sustainable population size to order 1 (individuals, households, village clusters, depending on the spatial scale of the habitat implied in the units of *x*_*i*_, etc.) *α* = 1.

Fig 4 illustrates the benefits of cycling through habitats that boom and bust at different times. In this case, we force habitat partitioning. This means that we force the agent from group one to spend 100 % of her time in habitat one and the same for group two in habitat two. At a low population density (the blue line in Fig 4), resource harvest always meets a forager’s target of calories (*h*_*m*_). It is only when scarcity emerges that the incentive to cycle on a seasonal time-scale becomes clear (shift from blue-to-yellow-to-red curves). At *N* = 3.5 (the red curve), for example, during lean periods, foragers work at the max tolerable level (12 hours per day) while falling far short of their calorie target (Fig 4b). This situation is untenable. Foragers must shift between habitats to maintain a consistent intake of calories.

**Fig 5:**
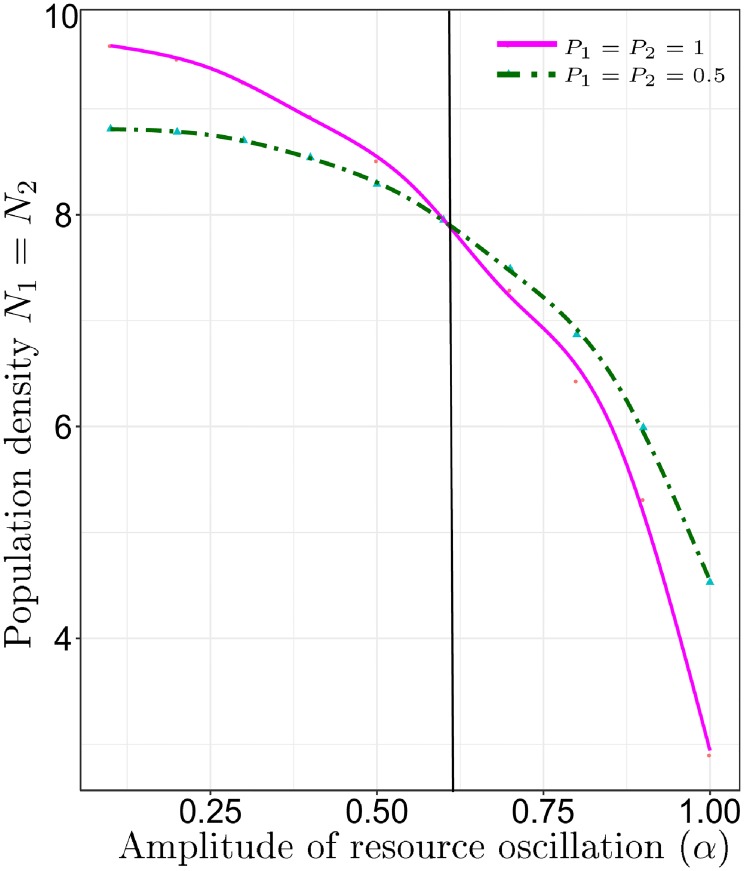
Maximum population density at which foragers can maintain a consistent diet that meets the target of *h*_*m*_ over 50 years when partitioned (*p*_1_ = *p*_2_ = 1, solid magenta line) and when mixed (*p*_1_ = *p*_2_ = 0.5, green-dashed-dot line). In high amplitude environments, migration between habitats has a higher maximum density than no migration, and in low amplitude environments, the opposite is true.

As illustrated in Fig 5, above a threshold of *α* = 0.58 in seasonality–the degree of resource boom-bust between winter and summer–forager cycling maximizes the population density at which foragers consistently meet their calorie target (*h*_*m*_) over 50 years. Below this seasonality threshold, habitat partitioning maximizes the population density at which foragers can meet their calorie target. The parameter space above the two curves includes population densities at which the model transitions into a degraded resource state. In this range of population densities, foragers cannot consistently meet their resource target, no matter what strategy they use. The parameter space below the curves includes population densities at which both strategies, forager cycling or habitat partitioning, allow foragers to consistently meet their calorie target. The lower a realized population density is relative to the maximum values defined by the curves in Fig 5, the wider the range of initial conditions at which partitioning and forager cycling are equivalent.

**Fig 6:**
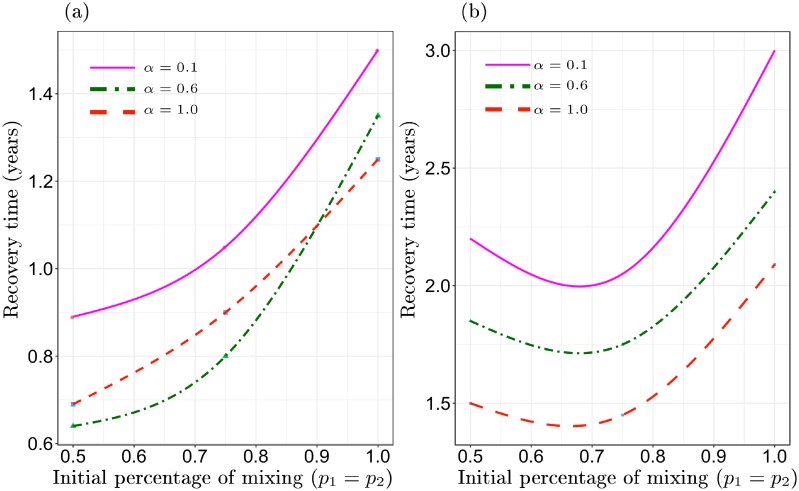
The recovery time (y-axis) it takes a population of foragers to consistently meet their calorie target following a perturbation to habitat one (a drought) for different levels of initial cycling (x-axis). Graph (a) population density is 1/4 of the maximum population density recorded in Fig 5 for a given level of resource oscillation (*α*). Graph (b) population density is 1/2 of the maximum recorded in Fig 5. In general, the graphics illustrate that full partitioning drastically increases recovery time from a perturbation.

However, Fig 6a & 6b illustrate a tradeoff associated with partitioning. The recovery time of foragers when a perturbation hits habitat one (e.g., an extended dry period) increases among partitioned foragers. The results in Fig 6a & 6b were generated by starting the initial biomass of habitat one at a very low level under two different population densities: One fourth the maximum density at which foragers meet their calorie target (Fig 6a) and one half of the maximum population density (Fig 6b). The x-axis records the initial degree of forager cycling (distribution of population in space), and the y-axis records the time (in years) that it takes foragers to recover from the perturbation to habitat one and converge to meeting their calorie target (*h*_*j*_ = *h*_*m*_). At both levels of population density, higher forager cycling results in a much faster recovery than more partitioning.

For example, in a low population density and aseasonal environment (solid magenta line, Fig 6a), a population of foragers that spends 50% of their time in each habitat recovers 3/4 of a year faster than a partitioned population. It would not take a very high frequency of such perturbations, perhaps one per decade, to increase fitness for foragers who cycle relative to those who partition. In sum, at small time-scales and low population densities, rapid forager cycling and partitioning seem equivalent, in terms of the consistency of calorie intake, but at larger scales forager cycling has a clear advantage. Even at low population densities and in unrealistically aseasonal environments, forager cycling increases the consistency and mean of harvests over time. In short, we should expect well mixed distributions of foragers in space and time, even in unrealistically aseasonal environments.

### Result 3: A variance reduction, safe-operating space tradeoff

**Fig 7:**
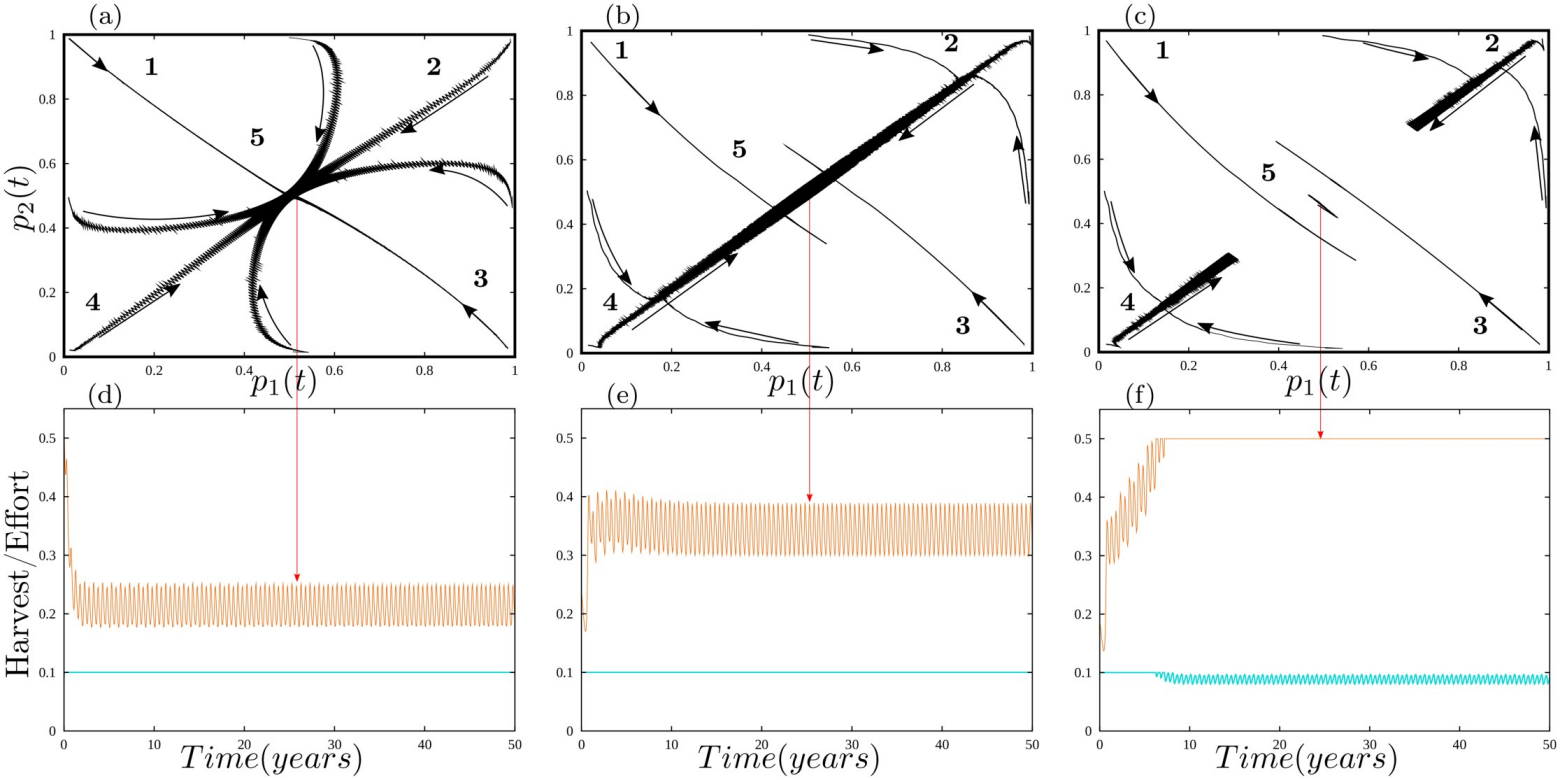
Phase plane analysis of spatial effort allocation. In all cases, *α* = 1. graphs a-c are phase plots that illustrate changes in the proportion of time allocated to habitats one and two over time by groups one and two for different initial conditions. In graph (a) *N*_*i*_ for all i = 2.5; in graph (b) *N*_*i*_ for all i = 4.25; in graph (c) *N*_*i*_ for all i = 4.5. Graphs d-f illustrate total foraging effort (*e*_11_ + *e*_12_, orange curves) and the calorie harvest (*h*_1_-blue curve) for the forager in group one as the system converges to a perfectly mixed equilibrium, on average, of *p*_1_ = *p*_2_ = 0.5. Graphs d-f illustrate the mean hours of work a forager from group one must put-in to gain food and the consistency of hitting the calorie target (*h*_*j*_ = *h*_*m*_) over time when foragers from both groups perfectly mix in space. The dynamics are analogous for the forager from group two.

Fig 7 displays a powerful variance reduction, safe-operating space tradeoff associated with forager cycling. Fig 7a–7c display three phase plots. Phase plots display the relationship between variables that change over time. For example, in Fig 3a the biomass of habitats one and two vary over time and are 180 degrees our of phase (i.e., when one peaks, the other troughs). An alternative way to plot this relationship is in phase space, plotting the biomass of habitat one on the x-axis, and the biomass of habitat two on the y-axis. In this example, the graph would display a perfect negative correlation. Imagine a movie of a pencil drawing this relationship on the graph. The pencil would start in the lower right corner (habitat one high biomass, habitat two low biomass) and, over time, move along a perfect linear trajectory toward the upper left corner of the graph and then back to the bottom right corner. The pencil will just oscillate along this trajectory over time. Displaying how variables in a dynamical system relate to each other in a phase plot is a powerful tool for studying the coevolution of those variables over time and identifying emergent changes in the qualitative structure of a system under different parameter values.

The x-axis on each plot of Fig 7a—7c displays the proportion of time that group one spends in habitat one, and the y-axis displays the proportion of time that group two spends in habitat two. On each plot we mark five regions. Regions 1 and 3 are analogous to each other. In these regions, both foragers spend near 100 % of their time in the same habitat (Region 1, everyone in habitat two, and Region 3, everyone in habitat one). Regions 2 and 4 are, again, analogous to each other. In these regions, the system displays partitioning. In Region 2 the group two forager spends near 100% of her time in habitat two, and the forager from group one near 100 % of her time in habitat one. Region 4 displays the opposite. Finally, Region 5 displays mixing in space and over time. At the very center of this region foragers spend, on average, 50 % of their time in each habitat.

The black curves that traverse Fig 7a—7c illustrate the relationship between the proportion of time spent in habitats one and two as the system changes over time. The arrows along the black curves illustrate the direction of change over time. For example, imagine a system that starts at an initial condition in Region 1 on plot Fig 7a. In this case, the system starts with the foragers from groups one and two spending almost 100 % of their time in habitat two. The arrow pointing toward the middle of the graph indicates that, over time, the mean proportion of time each forager spends in each habitat converges to a constant in the middle of the graph, after which the system oscillates with constant mean around 0.5 (a perfectly mixed system with a forager spending 50 % of her time in both habitats). To give some intuition in terms of foraging populations, Fig 7 allows us to study how internal changes in the use of space, such as groups aggregating into habitat one for an important ceremony or groups completely partitioning to their associated ‘home-range’ affects the long-term evolution of the system under different population densities.

Fig 7a illustrates that when population density is low, the system is globally robust to changes in the use of space, including highly aggregated populations (e.g., *p*_1_ = 0.99, *p*_2_ = 0.01) or highly partitioned populations (e.g., *p*_1_ = *p*_2_ = 0.99). In the parlance of sustainable human-resource systems, the system has a complete safe-operating space. The safe-operating space includes all of the starting values of (*p*_1_, *p*_2_) on Fig 7a from which the system will evolve to a state where foragers consistently meet their calorie target.

Again, thinking intuitively about real foragers, consider the low population density case illustrated by Fig 7a. Observing an important ceremony requires that forager populations temporarily concentrate in space (e.g., in Region 1 of Fig 7a, foragers aggregate in habitat two). Due to this population concentration, foragers must work very hard to meet their energy target (i.e., effort is at 0.5 in Fig 7d at *t* = 0–the start of the ceremony). When the ceremony ends, foragers quickly move away from the extreme values in Region 1 toward Region 5, and foraging effort in habitats one and two quickly drop below the maximum tolerable work level and remain there. The orange curve on Fig 7d illustrates this dynamic for the forager from group one over time (the dynamic is analogous for the forager from group two). Initially, the orange curve is very high, then quickly drops and oscillates around a stable mean of 0.21 workdays/day, and the forager consistently meets her target harvest of calories (cyan line in Fig 7d) over time. In short, Fig 7a illustrates a system in which forager cycling minimizes variation in the calorie intake of foragers (at multiple scales as demonstrated above) *and* has a complete safe-operating space.

Fig 7b illustrates the consequences of increases in population density to near carrying capacity (here, *N*_1_ = *N*_2_ = 4.25 & *α* = 1). This increase has two consequences. First, in a perfectly mixed system (rapid forager cycling), foragers continue to meet their calorie target at equilibrium (cyan line is constant at 0.1 in Fig 7e), but at a lower rate of return. The orange curve in Fig 7e illustrates changes in foraging effort over time as the system converges to an equilibrium at *p*_1_ = *p*_2_ = 0.5. Note the higher mean of the orange curve in Fig 7e (≈ 0.35) than in Fig 7d. This means that the group one forager works harder to maintain a constant intake of calories.

Second, the increase in population density reduces the *p*_1_–*p*_2_ space in which foragers can converge to meeting their calorie target. Another way to say this is that the constant cyan curve in Fig 7e only emerges when the system begins from a mixed state. If foragers too unevenly distribute on the landscape either due to aggregating (both foragers spend most of their time in one habitat, such as Regions 1 and 3) or partitioning (each forager spends most of their time in one habitat, such as Regions 2 and 4), the system will converge to one that is mixed, but foragers experience long-term variation in their mean intake of calories (a result analogous to Fig 7f where the cyan curve oscillates). In this environment, the processes of aggregation or partitioning results in foragers not meeting their resource target. The safe-operating space of the system, the range of initial (*p*_1_, *p*_2_) values from which the strategy of cycling between habitats leads to a consistent supply of calories over the long-term, declines in size. If foragers become too aggregated or too partitioned, the consequence is oscillating resource shortfalls; a sequence of: Good year, shortfall year, good year, shortfall year, etc.

For instance, when the system starts with everyone aggregated in habitat two (Region 1), foragers must, again, start by working as hard as they can to harvest food. This depresses the productivity of habitat two. Because the cost to migrate is zero (*q*_*ij*_ = 1 for all *ij*), foragers head for habitat one, which depresses the resources in this habitat and causes foragers, again, to work as hard as they can. Over time, foragers converge back to a mixed system, but it is too late, in a sense, because the resource base in both habitats never has sufficient time to recover. Thus, foragers end up in a long-run equilibrium in which they can meet their resource needs every other year, but also experience a 10-20 % shortfall every other year. In sum, although forager cycling reduces variation in the calorie intake of individuals and raises the carrying capacity of many environments, the very success of the cycling strategy (leading to population growth until near carrying capacity) reduces the ability of foragers to aggregate and disperse without causing resources to vary unexpectedly as a consequence of their movements. This is a variance reduction, safe-operating space tradeoff. The very strategy used to reduce variation in the intake of calories leads to a decline in the size of the safe operating-space of the system. This is not something foragers could necessarily recognize unless they experienced the negative consequences.

Fig 7c illustrates a population density above the carrying capacities identified in Fig 5a (*N*_1_ = *N*_2_ = 4.55 & *α* = 1) and, in this case, there is no safe-operating space. All initial values of (*p*_1_, *p*_2_) lead to a situation in which foragers do not meet their calorie target, regardless of whether they cycle or partition. In the long-run, effort is maxed out (*e*_*i*_(*t*) = *e*_*x*_), and harvest is less than the preferred target (*h*_*j*_(*t*) < *h*_*m*_) for some time intervals in the annual cycle (Fig 7f). This opens up a region in *p*_1_ − *p*_2_ space that cannot be reached from all initial conditions (i.e., generates habitat partitioning). In this setting, the long run mean proportion of time spent in a given habitat depends on initial conditions, creating a barrier between Regions 2 and 4 because the capacity of the internal dynamics of the system to reach well mixed states is limited, and only external forcing (e.g., climate variation beyond normal seasonality) can temporarily mix the system.

The bottom line: foragers have overshot carrying capacity and entered a “Malthusian Purgatory.” No matter whether they cycle or not, they cannot meet their desired energy target. Thus, initial conditions determine the degree of partitioning. More technically, when foragers work as hard as tolerable, their choices about foraging in different habitats decouple from productivity across habitats. Under conditions of relative abundance (*e*_*i*_(*t*) < *e*_*x*_) foragers adjust effort to create a match between their calorie uptake and desired calorie uptake (move *h*_*j*_(*t*) toward *h*_*m*_). This decision impacts biomass (*x*_*i*_) which, in turn, impacts foraging effort (*e*_*i*_(*t*)), which starts the cycle anew. Thus, foraging effort (*e*_*i*_(*t*)) depends on both the proportion of time in a given habitat (*p*_*i*_) and biomass (*x*_*i*_), coupling their dynamics in such a way as the average over an annual cycle of *p*_1_ and *p*_2_ become equal. However, when foraging effort equals the maximum tolerable effort (*e*_*i*_(*t*) = *e*_*x*_), foraging effort *e*_*i*_(*t*) no longer depends on the proportion of time in a habitat (*p*_*i*_) and biomass (*x*_*i*_), it is simply a constant. This weakens the coupling between the proportion of time in a habitat and the biomass of a habitat. Thus, a mixed distribution of foragers in space bifurcates into a potentially partitioned distribution, depending on initial conditions.

### Territoriality

A qualitative insight with important consequences emerges from the comparison of Fig 7b and 7c. In the environment illustrated by Fig 7b, foragers are vulnerable to a cascade of oscillating resource failures. For example, if foragers respond to a drought in habitat one by aggregating in habitat two for a season, then in a system with free movement, this behavior generates a cascade of resource failures over the following years. But, the resource system still has enough stocked-up biomass that, if a bumper year hits in which productivity is above normal, the system could transition back into one in which individual foragers consistently meet their calorie target. Foragers can learn that their high cycling strategy is vulnerable and, thus, adjust their strategies to reduce the chances that they experience a sequence of resource shortfalls again.

In the environment defined by Fig 7c, foragers have crossed into a Malthusian Purgatory. Partitioning follows from overshooting the carrying capacity of the environment. The only way to deal with this is a massive migration or suffer prolonged negative fitness, until population is, again, well below carrying capacity and the resource base can recover. As Cowgill noted long ago, changes in strategy are unlikely in such a situation; rather depression and stagnation are likely because individuals lack adaptive capacity [43]. Partitioning in real systems is not likely a simple consequence of changes in equilibirum resource abundance and competition among foragers in different habitats. Rather, partitioning results from a complex interaction of resource abundance, the perception of foragers and the dynamics of knowledge creation as foragers move on a landscape.

**Fig 8:**
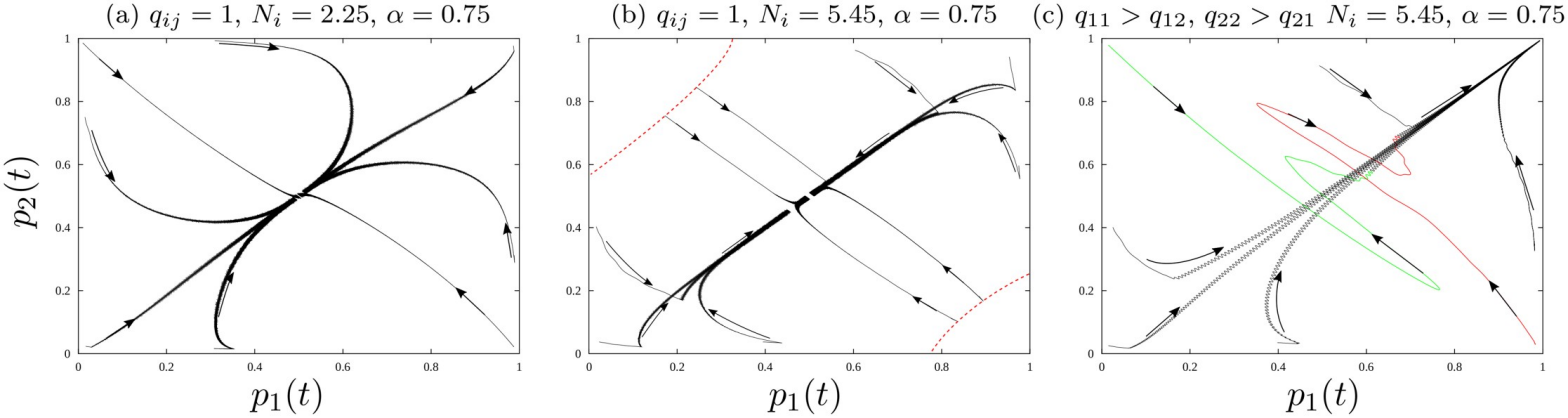
Phase plane analysis of spatial effort allocation; red curves illustrate the initial conditions in the *p*_1_ − *p*_2_ space in which foragers do not consistently meet their resource target *h*_*m*_, even though the system converges to a mixed state. As population density increases from (a) to (b), the initial conditions in *p*_1_ − *p*_2_ space from which foragers can consistently meet their resource target *h*_*m*_ declines. Graphs (c) illustrates the effect of territorial ownership. Territorial ownership enlarges the initial conditions in *p*_1_ − *p*_2_ space from which foragers can meet their resource target. In graphs (a)–(c), the amplitude of variation in *x*_1_ & *x*_2_ is held constant at *α* = 0.75. To define the red curves in Fig 7b, we ran 50 numerical experiments varying *p*_1_ and *p*_1_ holding all parameters equal and the initial conditions of *x*_1_ = 0.3 and *x*_2_ = 0.8 in all experiments.

To explore this dynamic further, Fig 8a–8c replicates Fig 7a–7c except in two crucial respects. First, foragers occupy a one quarter less seasonal environment, and, second, in Fig 8c we keep population density equal to Fig 7b and assume that there is a cost to enter another group’s home-range due to territorial norms. In particular, we assume that searching for resources within one’s home-range is free, but searching for resources outside of one’s own home-range has a cost (either avoiding attack or securing a ritual gift). In Fig 8a and 8b, we observe, again, that increasing population density reduces the *p*_1_–*p*_2_ space in which foragers can converge to meeting their resource target. In Fig 8a, again population density is low, and the safe-operating space of the system encompasses all potential values of (*p*_1_, *p*_2_). Increasing population density to the level in Fig 8b, decreases the safe-operating space of the system. The red curves on Fig 8b illustrate the safe-operating space. If foragers aggregate too far, (e.g. beyond the red curves), they will experience cascades of resource short fall that oscillate over time.

In this environment, territorial ownership has the consequence of enlarging the *p*_1_-*p*_2_ space in which foragers can converge to meeting their calorie target, though the system now always converges to a partitioned state. Fig 8c introduces a cost for group one to access habitat two and for group two to access habitat one. This cost slows the flow of foragers between habitats in response to differences in harvest per unit effort. In turn, as foragers spend more time in their own habitat, they loose information on the other habitat and discount traveling there relative to harvesting resources from their own ‘home-range.’ This dynamic leads to the complete partitioning of groups in space and time. Such partitioning based on territorial ownership cannot happen when *α* = 1 (very high seasonality) because partitioning creates too much concentrated harvest pressure on a given habitat. However, where *α* = 0.75 ownership norms allow individuals to consistently meet their resource target, whether partitioned or highly aggregated. Foragers can partition because the resource base in each habitat can withstand more intense harvest and foragers can aggregate because territorial ownership prevents a massive swing of population from one habitat to another, slowing down the movement of foragers on a landscape. The adoption of territorial ownership, in an emergent way, stabilizes the harvests of individual foragers by reducing the risk of leaving a system’s safe-operating space.

## Discussion

Our analysis of the SPDm allows us to explore the question posed in the introduction: What potential mechanisms drove habitat partitioning on the TCP? A powerful way to gain insight into the processes that may lead to rapid cycling vs. partitioned populations of foragers is to construct a dynamic ecological model of hunter-gatherer habitat use that we call the SPDm. With the SPDm, we can study the feedback between decisions about how much time to spend in a given habitat and how many habitats to use. Similarly, we modify the assumptions, typical of IDMs and the MVT, that foragers have complete information on the resources among alternative habitats on a landscape and that mean resource abundance is stable over time. These modifications move the SPDm closer to the reality that forgers face without attempting to replicate all of the decisions that real foragers face in particular environments. Thus, the SPDm, though more complex than IDMs and the MVT, retains some generality.

The key result of our analysis is the existence of a variance reduction, safe-operating space tradeoff. This tradeoff is a special type of tradeoff under the more general umbrella of robustness-fragility tradeoffs. This type of tradeoff emerges from individual decisions about the use of habitats and the responses of individuals to changes in resource density. Forager cycling between habitats stabilizes the intake of calories for individuals in the short-run, but the very success of this adaptation may lead to population growth and the emergence of vulnerability to climate change and internal social dynamics in the longer-run (a reduction in safe-operating space). Robustness-fragility tradeoffs commonly occur in social-ecological systems [32, 33, 44–49], and, we argue, such tradeoffs are under-appreciated mechanisms that drive social change. Robustness-fragility tradeoffs do not determine the form of social change, but set the preconditions for shifts in strategies for interacting with the environment.

For example, our analysis illustrates that the severity of seasonality and population density interact on smaller time-scales. When population density is low, foragers may partition or cycle between habitats. The two strategies are equivalent (Figs 4 and 5), if the goal is to maintain a stable supply of calories. Of course, in a low population density environment other incentives beyond a consistent supply of resources at a seasonal time-scale exist to cycle, like finding mates. Similarly, even in aseasonal environments, climate perturbations, like droughts that occur at decade frequencies, generate an incentive to cycle for individuals (Fig 6). The Texas Coastal Plain is a temperate environment with a moderate degree of seasonality. Thus, we would expect, at low densities, climate variation on seasonal and decade scales to favor high cycling (lots of visitation, shifting of group residence and so forth). However, if population density were to increase relative to resources at a landscape level (multiple habitats), forager cycling would insure a consistent diet, but would also cause the safe-operating space of the whole system to decline.

**Fig 9:**
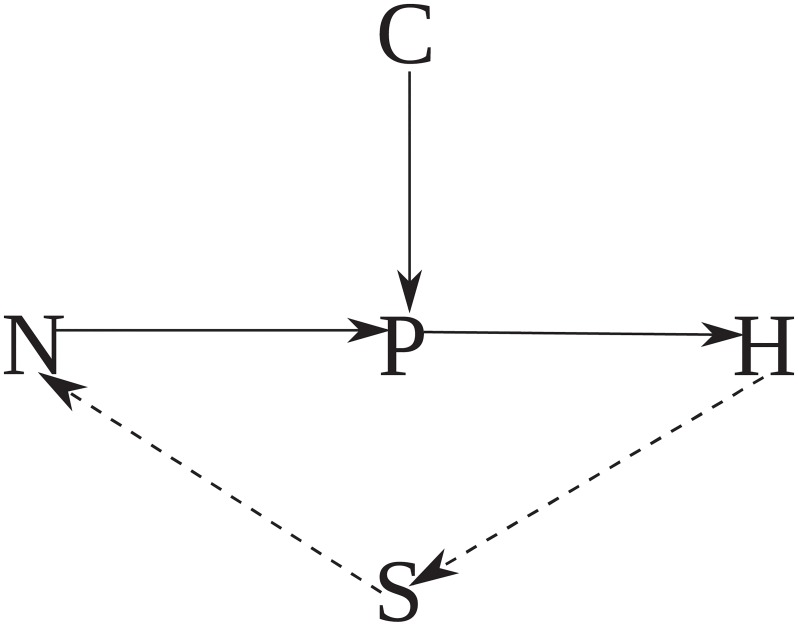
Proposed causal diagram of the factors that lead to partitioning. N = Population density, P = productivity, C = climate variation, H = Harvest, and S = Strategy.

Fig 9 summarizes a proposed causal process for the TCP. Population (N) and Climate (C) affect the productivity of resources (P). Foragers apply knowledge to harvest resources and generate a harvest (H) of calories. Harvest feeds back to population, mediated by the strategy (S) used by foragers to monitor the flow of foragers on a landscape (open access, free to cycle vs. ownership, higher cost to cycle). In this loop, forager cycling leads to consistent harvests, in spite of climate shocks, which leads to population growth and, in turn, growth affects productivity and leads to the variance reduction, safe-operating space tradeoff. Two mechanisms might lead to partitioning. The first is descent into Malthusian Purgatory (Fig 7c). In this case, population would overshoot carrying capacity and the system of land use would bifurcate into one in which foragers work 12 hours per day wherever they happen to locate. No incentive exists to move. As noted earlier, there would also be very little incentive to adopt territoriality in this situation as it would mean a lot of effort just to maintain a potentially less negative level of fitness. Fitness would still be negative, however. Population migration and long-term depression should follow such a Malthusian overshoot.

Second, climate variation may generate a ‘signal’ to individual foragers [32] that they should adapt to stabilize their flow of resources. One form of adaption is territorial ownership, which would lead to habitat partitioning. The role of information in moderating decisions about the proportion of time to spend in any given habitat underlies this mechanism. Recall, rapid cycling creates a more consistent intake of calories and raises the carrying capacity of human foragers on a landscape (Figs 3 & 5), in part through the acquisition of information that makes accessing resources more efficient. Indeed, if foragers were to engage in the exchange of goods and form networks on top of local kin based relationships, this would generate even more return from cycling. Knowledge infrastructure would generate ever more efficient flows of information and, at least initially, as population density increased foraging would become more efficient [50]. Thus, incentives en-train rapid cycling in foraging economies, even among populations living in aseasonal environments and undergoing increases in population density. The trick to understanding the emergence of partitioning is understanding that even though a rapid cycling strategy is resistant to increases in population density, the safe-operating space of the whole system declines.

A decline in safe-operating space proves a paradox. Reductions in the safe-operating space set foragers up for cascades of resource failure. However, such a process also provides a potential Boserupian escape from Malthusian overshoot. In terms of the TCP, one might imagine a spurt of sea-level rise along the coast that inundates estuaries with salt water. This would depress productivity for a few years as species adjust their ranges and so on, stimulating foragers to aggregate among inland habitats (e.g., Region 1 on Fig 7). At low population density, this strategy works. However, as the system approaches carrying capacity and the safe-operating space declines, this behavior would generate resource short-falls that oscillate over time generating a successful year, bad year, successful year, etc. sequence. This very pattern provides a signal that the knowledge infrastructure so integral to cycling between habitats is worthless. The incentive thus arises for foragers to shift their social norms of land use, generating partitioning in an emergent way on the landscape. Crucially, in this scenario the resource system still has enough biomass capital relative to population size that, if a bumper year of productivity hits, the system could transition back into one in which individual foragers consistently meet their calorie target. The capacity exists for foragers to learn and adjust their strategies to reduce the chances that they experience a sequence of resource shortfalls again.

The above scenario suggests a set of potential predictions in archaeological contexts.

1. We should expect population regime changes on the TCP or in any environment in which hunter-gatherers shift to a partitioned land use system. The term regime change here has a specific meaning. It means that the parameters that control a human population (e.g., r and K in the logistic model), should display large, sudden changes in a time-series. One way to begin to evaluate this prediction is to analyze large radiocarbon data sets and evaluate the null hypothesis that the statistical distribution of radiocarbon is constant over time. If a demographic system experiences a regime change, the amount of radiocarbon should display multiple means. This would be consistent with the scenario above that partitioning emerges quickly as foragers adopt territoriality and more intensive subsistence practices as a package, leading to a punctuated increase in mean population density on the landscape.
2. Coincident with a regime shift in the radiocarbon record, we should observe a change from passive, pervasive exchange systems to spatially bounded reciprocal exchange between distinct cemetery populations. As noted in the introduction, the TCP has a 7,500 year burial record. We should observe early on in that record, a spatially unrestricted exchange as individuals cycle on a landscape and develop wide ranging connections. However, when partitioning emerges, the movements of foragers should be much more tightly bound in space to areas in which resources negatively correlate in time (when one resource is up, the other is down), and reciprocity should be intensive as territorial boundaries have become more rigidly enforced, and reciprocal gifting is a key way to gain entry across boundaries. To evaluate this idea, we would need a data set of grave goods associated with individuals and tight temporal control over those individuals. Similarly, if we were to track gene flow via aDNA, we might expect to see a drop in genetic diversity as regional exchange systems become more tightly bound in space (if mates and status/wealth items follow similar flows).
3. Coincident with the above patterns, we should see an increase in the ^13^C and ^15^N bone isotope diversity displayed among individuals. High cycling means more mixing of individuals in space, and thus across resource zones. This should lead to individuals that, over time, have more similar looking diet profiles in their isotopic signatures. Conversely, partitioning (low cycling) means that individuals more spatially restrict their use of resources. In the scenario above, individuals restrict to particular habitats that they maintain more exclusive control over and, thus, their diet should reflect more the isotopic signatures of the local ecology that they exploit more often. To evaluate this, we would need ^13^C and ^15^N bone isotope data on a large number of individuals over the full 7,500 year period of burials on the TCP. Coincident with a demographic regime shift noted above and more spatially defined grave goods exchange, we should see a spike in the diversity of isotopic signatures among individuals. Finally, strontium isotope studies (^87^Sr/^86^Sr) on human teeth have potential for evaluating expectations from the model, assuming regions with contrasting levels of bioavailable strontium isotopes are used. During periods of more rapid cycling, samples from a number of mortuary sites from different regions should show greater similarity among samples and should have greater internal variability as individuals move among regions. As partitioning emerges, then variability within a sample should decline and greater differences among samples should be detectable.

## Conclusion

In this paper, we have proposed an ecological theory of mobile hunter-gatherer population distribution, and made this theory operational with a spatial population distribution model (SPDm) built to answer a specific question: What mechanisms might drive the evolution of habitat partitioning? The Marginal Value Theorem and Ideal Distribution Models serve as a starting points for our investigation. These models drawn form Foraging Theory investigate, separately, crucial processes that affect hunter-gatherer land use and degree of partitioning. The SPDm adds a layer of realism to these general models by investigating the relationship between habitat choice and time within a habitat as inseparable from the response of resources to external climate drivers and the harvest behavior of other individuals on a landscape. We argue that major changes in the distributions of hunter-gatherers relate to the efficacy of different strategies for maintaining a consistent supply of resources. Cycling among habitats and social groups is a form of cooperation dependent upon building diverse social networks and sharing information. A rapid forager cycling equilibrium is highly resistant to increases in population density, and should only change as foragers find the flow of information about the quality of habitats on a landscape to degrade and must rely more on their own and cooperative labor in more local contexts to maintain a consistent flow of resources.

Potential directions for future research include:

1. Rigorously distinguish between population overshoot and collapse vs. a system that experiences a disruption from crossing a safe-operating space boundary, but the individuals still have the capacity to adapt and find new strategies that fundamentally change the system. A true Malthusian system would not experience a regime shift to a higher carrying capacity system, rather the functional relationships between variables would not change. Conversely, our argument for territoriality would imply a fundamental regime shift in the relationship between population growth, carrying capacity and land use. Detecting regime shifts in empirical time-series is a growing area of research and a key to distinguishing between these two scenarios [51–53].
2. A second area of research concerns middle range research on the potential for multiple regimes among ethnographically recorded hunter-gatherers [52]. We should observe fundamentally different relationships between land use and population among ethnographically documented hunter-gatherers due to partitioning generated by the adoption of territorial norms. The fundamental change in ownership norms should change the way that information moderates land use.
3. We have only begun to explore the potential of the SPDm as a general framework for understating the use of space by foraging populations over time. For example, we could investigate the effects of an increasingly patchy environment on the dynamics of the model. This is easily done with the current model by increasing the travel costs between patches. This is just one example of a rich number of dynamics that may be explored with the model.

## Model and methods

Formally, the resource dynamics of the SPDm are given by

$${\dot{x}}_{1}\left(t\right)=G_{1}\left(x_{1}\right)-dx_{1}-H_{1}$$ (1)

$${\dot{x}}_{2}\left(t\right)=G_{2}\left(x_{2}\right)-dx_{2}-H_{2}$$ (2)

where the change in the abundance of resources in habitat one (${\dot{x}}_{1}$) is the growth of the resource (*G*_1_(*x*_1_)) in habitat one less the natural decay *dx*_1_, less the total biomass harvested by foragers, *H*_1_. The total harvest in habitat *i* is the sum of the harvests from each group. Table 1 summarizes the model’s state variables and parameters.

The growth of resources in each habitat, *G*_*i*_(*x*_*i*_) is defined by the logistic function *G*_*i*_(*x*_*i*_) = *x*_*i*_*r*(1 − *x*_*i*_/(*K*_*i*_ + *I*_*i*_)); where *r* is the growth rate of a resource base, and scales the response of a given ecosystem to an external driver, which scales the variation of the resource, and *K*_*i*_ is the initial carrying capacity of a resource base. In a two habitat model, we have two inflows (external drivers), *I*_1_ and *I*_2_. These inflows represent an influx of energy or water that seasonally change the carrying capacity, *K*_*i*_ of a resource base, and these inflows are sinusoids with a mean of 1. For example, if *I*_1_ = 1 + *α* sin(2*πt*) and *I*_2_ = 1 − *α* sin(2*πt*), then *I*_1_ and *I*_2_ are 180 degrees out of phase, which may represent summer–winter peaks and valleys in the availability of resources, respectively; while *α* is the amplitude of the peaks and valleys (Fig 3a).

**Table 1:** Model state variables and parameters.

**State Variables**	**Definitions**
*x*_*i*_(*t*)	The density of resources (biomass/area) in habitat i at time *t*
*p*_*i*_(*t*)	The proportion of effort spent in habitat i at time *t*
*e*_*ij*_(*t*)	The effort spent in the harvest of resources in habitat i by group j at time *t*
**Parameters**	**Definitions**
*α*	The amplitude of resource variation (the severity of boom-busts)
*r*	The mean intrinsic rate of resource growth (yr^−1^)
*K*_*i*_	The maximum abundance of resources in habitat *i*
*I*_*i*_	The inflow of energy into habitat i
*d*	The natural decay of resources
*q*_*ij*_	The transaction cost of accessing habitat i for group j
*N*_*j*_	The population of foragers in group j
*h*_*m*_	The energy target per forager
*r*_*p*_	The strategy adjustment rate

The variable *H*_*ij*_ is the total harvest from habitat *i* by group *j* (*H*_11_, *H*_21_, etc.). We define the harvest of an individual as *h*_*ij*_ = *q*_*ij*_*e*_*ij*_*x*_*i*_, where *e*_*ij*_ is the effort an individual from group *j* spends harvesting in habitat *i*. The parameter *q*_*ij*_ defines the “harvestability” of the resource per unit of resource, per unit of effort of an individual from group *j* in habitat *i*. Total harvests are, then, individual harvest multiplied by population size: *H*_*ij*_ = *N*_*j*_*h*_*ij*_, where *N*_*j*_ is the population size of group *j*. The parameter *q*_*ij*_ scales (sets the units on) the sustainable population size of a given habitat. We set, initially, *q*_*ij*_ = 1 for *i* = *j* and *q*_*ij*_ = *q*. This means that there is no cost to access resources within habitats or between habitats, either in terms of mobility or participating in a gift exchange ceremony to access a habitat (e.g., [38]). The notion of territorial rights can be operationalized mathematically as *q*_11_ > *q*_12_ and *q*_22_ > *q*_21_. Under this condition, it is more costly for an individual in group one to enter the home-range of group two than forage in their own home-range.

Two basic assumptions guide how we model foraging effort (time spent harvesting food within a habitat). The first is that there exists some social convention that links individuals (kin or networks of kin) and that these social conventions are tied to home-ranges via a shared knowledge system [30]. Second, because we are interested in aggregate-level organizational patterns, we keep the individual model of decision making ecologically bounded. We model a representative agent that wants to meet their minimum harvest target, *h*_*m*_. Once this is met, foraging effort stops. A reasonable assumption among foragers who do not store food [54].

We assume that foragers attempt to meet a target resource uptake rate with a minimum expenditure of labor because this maximizes the time available for other fitness enhancing activities. Fig 3b shows the target resource level (the red curve) and the effort level necessary to achieve a constant resource target (black curve) for a partitioned forager in habitat one. When resources are scarce during the winter (blue curve in Fig 3a is at a minimum), effort is at a maximum. The dynamic is: Partitioned foragers adjust their work load to compensate for variation in the resource and, by doing so, maintain a constant intake of food.

In cases where variation in the availability of resources occurs out of phase, foragers may have incentives to migrate, in addition to adjusting their habitat specific foraging effort, to a habitat with more abundant resources to smooth out variation in their effort and returns from labor. To investigate this dynamic, we let *e*_*j*_ represent the total harvest effort of a representative agent from group *j*, and *p*_*j*_ the proportion of time individuals in group *j* spend in their “home-range.” Thus,

$$\begin{array}{cccc} e_{11}=p_{1}e_{1},\;\;\; & e_{21}=\left(1-p_{1}\right)e_{1},\;\;\; & e_{12}=\left(1-p_{2}\right)e_{2},\;\;\; & \;e_{12}=p_{2}e_{2}. \end{array}$$ (3)

Where *e*_11_ is the effort (time) expended to harvest resources in habitat one by group one, which is the effort expended on foraging by group one multiplied by the proportion of time spent in habitat one (*p*_1_). The term *e*_21_ is the effort expended to harvest resources in habitat two by group one and so on.

The harvest per unit effort in each habitat depends on resource abundance and the transaction costs associated with gaining access to those resources, which, holding effort equal, was represented above as: *h*_*ij*_ = *q*_*ij*_*x*_*i*_. Where *q*_*ij*_ is, as defined above, the harvestability of resources in habitat *i* by group *j*. Thus,

$$h_{1\;}\;=\;e_{11}h_{11}\;+\;e_{21}h_{21}$$ (4)

$$h_{2\;}\;=\;e_{12}h_{12}\;+\;e_{22}h_{22}$$ (5)

where *h*_1_ and *h*_2_ are the total harvests of a representative agent in group one and two, respectively.

An individual’s strategy about how much effort to expend (*e*_*j*_) and how to divide effort between habitats (*p*_*j*_) adjusts according to the following heuristic: First, “Do I feel hungry (is *h*_*j*_ < *h*_*m*_)?” If so, increase *e*_*j*_ until either *h*_*j*_ = *h*_*m*_ or foraging effort reaches a maximum tolerable level *e*_*x*_. Assuming that the decision to adjust foraging effort occurs on a smaller time-scale (days-months) than migration decisions (months-years), we formalize the change in foraging effort as an instantaneous process relative to the proportion of time spent in either habitat, and, thus, represent changes in foraging effort algebraically. That is, given *p*_*j*_, agents choose *e*_*j*_ such that *h*_*j*_ = *h*_*m*_ subject to constraint *e*_*j*_ < *e*_*x*_,

$$e_{1}=\min(\frac{h_{m}}{p_{1}h_{11}+\left(1-p_{1}\right)h_{21}},e_{x})$$ (6)

$$e_{2}=\min(\frac{h_{m}}{\left(1-p_{2}\right)h_{12}+p_{2}h_{22}},e_{x}).$$ (7)

Eqs (6) and (7) state that each representative agent minimizes their foraging effort in a given habitat to obtain their desired level of calories, unless this exceeds the maximum tolerable effort (*e*_*x*_).

Second, “Does changing my proportion of time in a habitat (*p*_*j*_) affect my harvest?” That is, is $h_{j}^{\prime }\left(p_{j}\right)$ positive or negative? If it is positive, then a forager is more willing to change *p*_*j*_. Willingness to change, however, also depends on how well a forager knows the other habitat. If *p*_*j*_ is high, foragers do not know much about the other non-home-range habitat. If *p*_*j*_ is low, foragers have been away from their home-range a lot, and don’t know it as well. This uncertainty reduces willingness to change *p*_*j*_. When *p*_*j*_ is an intermediate value (e.g., 0.5), foragers spend roughly equal time in both habitats, know both well, and are more willing to alter their existing strategy. The simplest way to say this is that information moderates decisions about whether to change habitats, which are made based on resource differences.

**Fig 10:**
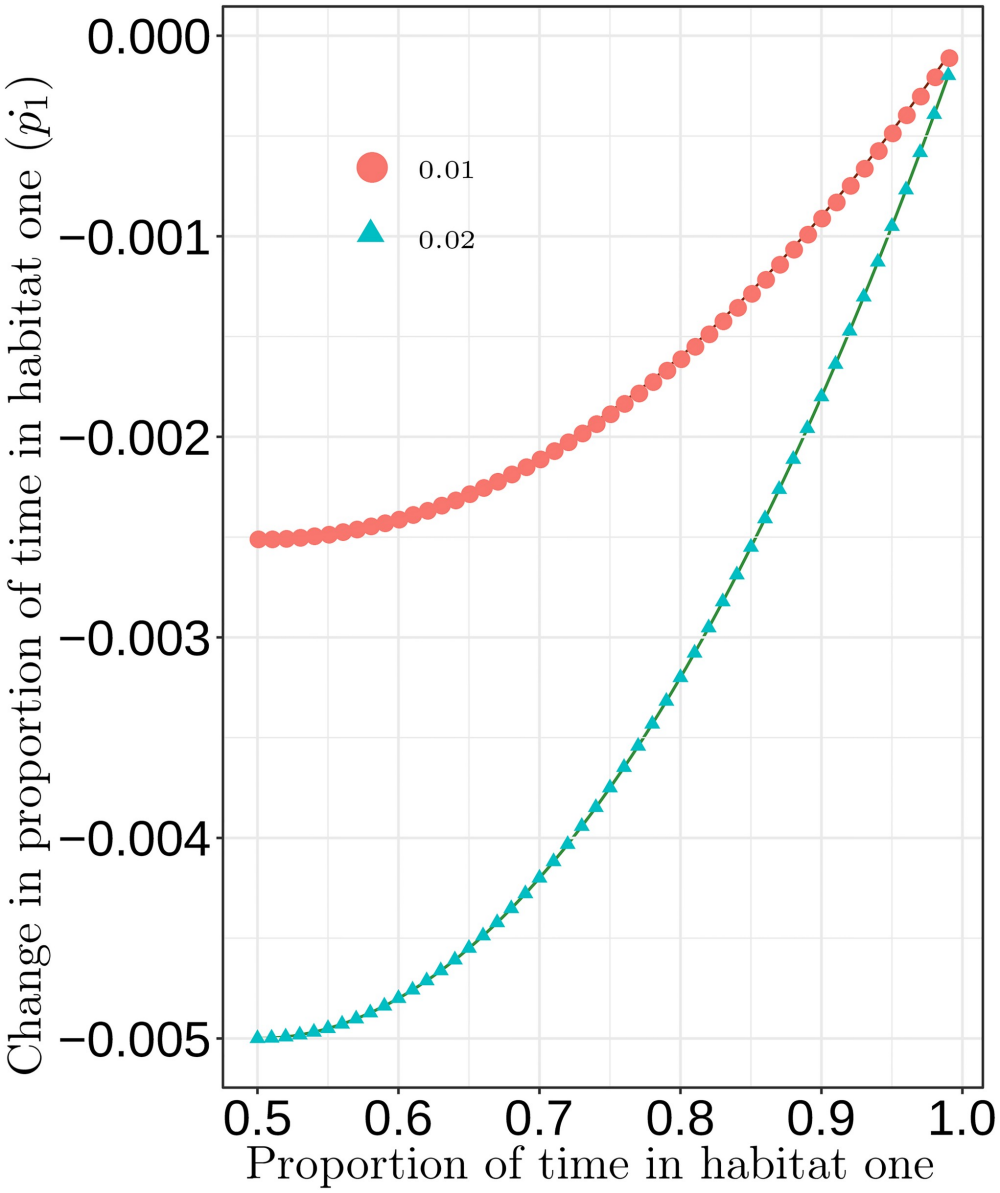
Illustration of how the proportion of time in a habitat moderates the effect of normalized differences in harvest per unit effort upon the change in proportion of time devoted by the group one forager to habitat one. In this case, harvest per unit effort is higher in habitat two than habitat one at two constant levels: 0.01 (orange dots) and 0.02 (blue triangles). Thus, the group one forager decreases the proportion of time spent in habitat one (y-axis), and this is moderated by the current balance of time spent in habitat one (x-axis). As the proportion of time spent in habitat one decreases, the forager discounts the potential value of resources in habitat two less and adjusts her balance of time away from habitat one more strongly.

There are a number of specific ways the comparison between habitats could be made by foragers. To avoid the problem of the overall scale of resource productivity impacting the comparison between habitats, we normalize by the average background productivity per unit effort. This is similar to the Marginal Value Theorem in which a forager compares their current return rate with the overall mean of all potential habitats in an environment. That is, let

$$\begin{array}{c} {\bar{h}}_{j}=\frac{h_{1j}+h_{2j}}{2} \end{array}$$ (8)

where the average harvest of group j equals their harvest in habitats one and two dived by two. Thus, adjustments in *p*_*j*_ are described by

$${\dot{p}}_{1}=B_{1}e_{1}\left(h_{11}-h_{21}\right)/{\bar{h}}_{1}$$ (9)

$${\dot{p}}_{2}=B_{2}e_{2}\left(h_{22}-h_{12}\right)/{\bar{h}}_{2}$$ (10)

where *B*_1_ and *B*_2_ are the balance of time currently spent in both habitats for the representative agent from group one and two respectively. More formally, *B*_1_ = *r*_*p*_(*p*_1_)(1 − *p*_1_), and *B*_2_ = *r*_*p*_(*p*_2_)(1 − *p*_2_) where *r*_*p*_ is the strategy adjustment rate. When *r*_*p*_ is higher, a forager adjusts to differences in normalized harvest per unit effort more strongly, placing less importance on information as *r*_*p*_ increases. Here we set *r*_*p*_ = 1. Fig 10 provides an illustration of the moderating effect of the balance of time spent in habitat one on how the forager from group one responds to normalized differences in harvest per unit effort between habitats one and two. In this case, we hold the difference in harvest per unit effort constant at two values: Habitat two provides a 0.01 and 0.02 greater harvest per unit effort than habitat one. Thus, the forager from group one should decrease the proportion of time they spend in habitat one ($\dot{p_{1}}$). The main point is that when the balance of time between the two habitats is equal at 0.5, a forager more quickly decreases the proportion of her time spent in habitat one because information is abundant on habitat two. However, as the proportion of time in habitat one approaches 1, the forager more strongly discounts the potential for resources in habitat two due to uncertainty about those resources. Thus, a smaller change in the proportion of time spent in habitat one.

### Methods of analysis

We analyze the SPDm numerically in XPPAUT [55], which is specialty software for analyzing non-linear dynamic systems models, and report our results at two different time scales. The smaller-time scale illustrates the mechanisms that drive the behavior of the model and subtle tradeoffs associated with rapid cycling through multiple habitats vs. habitat partitioning. The larger time-scale illustrates the conditions under which rapid cycling and habitat partitioning should emerge from individual decisions about how to best maintain a consistent supply of food in a variable environment. Our analysis of the larger-time scale focuses on two different perturbations that affect foraging economies: (1) declines in the availability of resources due to, for instance, a dry period, and (2) the process of periodic aggregations of foragers for important ceremonies or due to a climate perturbation. We used XPPAUT to run simulations and generate the phase plots illustrated in Figs 7 and 8. We used R to make simple graphs of the relationship between model parameters and simulated outcomes.

## Supporting information

S1 Appendix SPDm model code with comments to run using XPPAUT. (TXT)
